# Peroxiredoxin, Senescence, and Cancer

**DOI:** 10.3390/cells11111772

**Published:** 2022-05-28

**Authors:** Mengyao Wu, Chujun Deng, Tak-Ho Lo, Ka-Ying Chan, Xiang Li, Chi-Ming Wong

**Affiliations:** Department of Health Technology and Informatics, The Hong Kong Polytechnic University, Hong Kong, China; mengyao.wu@connect.polyu.hk (M.W.); 19048523r@connect.polyu.hk (C.D.); takho.lo@connect.polyu.hk (T.-H.L.); kaying007.chan@polyu.edu.hk (K.-Y.C.); xiang1.li@connect.polyu.hk (X.L.)

**Keywords:** peroxiredoxin, aging, cancer, oxidative defense system, redox signaling, chaperone, cycle cell progression, DNA integrity, inflammation, carcinogenesis

## Abstract

Peroxiredoxins are multifunctional enzymes that play a key role in protecting cells from stresses and maintaining the homeostasis of many cellular processes. Peroxiredoxins were firstly identified as antioxidant enzymes that can be found in all living organisms. Later studies demonstrated that peroxiredoxins also act as redox signaling regulators, chaperones, and proinflammatory factors and play important roles in oxidative defense, redox signaling, protein folding, cycle cell progression, DNA integrity, inflammation, and carcinogenesis. The versatility of peroxiredoxins is mainly based on their unique active center cysteine with a wide range of redox states and the ability to switch between low- and high-molecular-weight species for regulating their peroxidase and chaperone activities. Understanding the molecular mechanisms of peroxiredoxin in these processes will allow the development of new approaches to enhance longevity and to treat various cancers. In this article, we briefly review the history of peroxiredoxins, summarize recent advances in our understanding of peroxiredoxins in aging- and cancer-related biological processes, and discuss the future perspectives of using peroxiredoxins in disease diagnostics and treatments.

## 1. Introduction

Aging is an inevitable detrimental change caused by accumulation of damaged biomolecules that trigger dysregulation of cellular processes such as apoptosis, genome stabilization, and metabolism [1]. These dysregulations may eventually lead to cell transformation and tumor initiation [2]. That is the reason why aging is an important risk factor for cancers [3]. The risk for almost all cancers increases steadily with age. According to the National Cancer Institute Surveillance, Epidemiology, and End Results (SEER) Program 21, incidence rates of cancers increase dramatically from 25 cases per 100,000 people in people under 20 to over 1000 cases per 100,000 people in age groups over 60. Worse still, elderly cancer patients usually have much lower recovery rate and higher mortality. As the lifespan of worldwide population keeps increasing, there is an urgent need to develop novel strategies for cancer prevention and therapy in the elderly.

There are various protective mechanisms in our body to prevent accumulation of potential carcinogenic substances and to repair damaged macromolecules [3,4,5,6,7]. For example, various antioxidant enzymes are evolved to remove free radicals. DNA damage repair (DDR) pathways such as nucleotide excision repair (NER) and base excision repair (BER) function to fix oxidized DNA [8]. Moreover, there are proteasomes to remove oxidized proteins [9]. In this article, we first briefly review the history of identification and characterization of a ubiquitous multifunctional enzyme family named as peroxiredoxins. Then, we summarize the recent advances in our understanding of the function and role of peroxiredoxin in cancer- and aging-interconnected biological processes. Finally, we discuss the future perspectives of using peroxiredoxins in disease diagnostics and treatments.

## 2. Identification of Peroxiredoxins as Antioxidant Enzymes

The first peroxiredoxin was identified in budding yeast *Saccharomyces cerevisiae* as a sulfur radical scavenger in 1987 [10]. It was named thiol-specific antioxidant (TSA) at that time as this antioxidant enzyme required thiol for its activity [10]. Peroxiredoxins display several characteristics different from classical antioxidant enzymes. Firstly, they do not require any redox cofactors for function (such as copper/zinc for superoxide dismutase and heme for cytochrome c peroxidase). Secondly, a unique peroxidatic cysteine in the catalytic center of peroxiredoxins is responsible for their basic functions in oxidant defense [11]. Thirdly, these antioxidant enzymes can reduce a broad spectrum of oxidants including reactive oxygen species (ROS) and reactive nitrogen species (RNS) via the evolutionarily conserved redox-active cysteine [12,13]. These thiol-dependent peroxidases can be found in all organisms, and multiple isoenzymes are commonly found in one species. In 2016, this large and highly conserved family of peroxidases was renamed as peroxiredoxin by the Nomenclature Committee of the International Union of Biochemistry and Molecular Biology.

## 3. Classification of Peroxiredoxins

**Figure 1:**
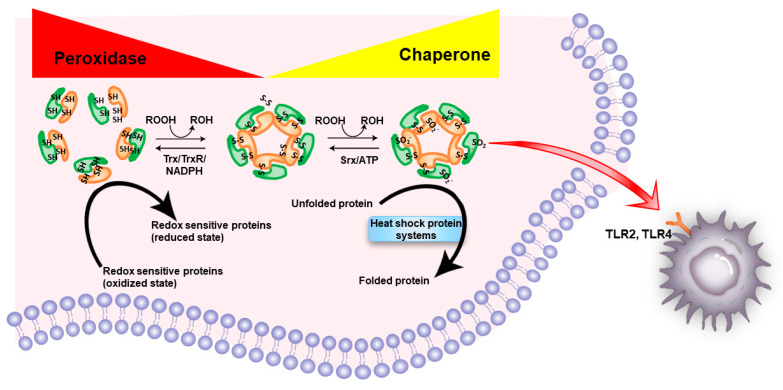
Schematic illustration of the general functions of peroxiredoxins. The highly conserved redox-sensitive cysteine residue (peroxidatic cysteine) in peroxiredoxin directly reduces various peroxide substrates (ROOH). A resolving cysteine can regenerate the peroxide reduction activity of the peroxidatic cysteine by thioredoxin (Trx)/thioredoxin reductase (TrxR)/nicotinamide adenine dinucleotide phosphate (NADPH) system. The peroxidase activity of peroxiredoxin can be easily inactivated by hyperoxidation of the peroxidatic cysteine to cysteine sulfinic acid (Cys-SO_2^−^_). A special oxidoreductase sulfiredoxin (SRX) is required to restore the peroxidase activity of hyperoxidized peroxiredoxins by reducing sulfinic acid of peroxiredoxin back to thiol in an ATP-dependent manner. Interestingly, hyperoxidated peroxiredoxin is required for the recruitment of cytosolic molecular chaperone HSP70 and disaggregase HSP104 to rescue misfolded proteins from aggregates. Extracellular peroxiredoxins function as DAMPs by triggering a proinflammatory response via binding to TLR2/TLR4 receptor.

As the amino acid sequences of the active sites of peroxiredoxins are highly conserved from bacteria to human, many new family members were identified by sequence homology in the last decade [14]. The current classification system classifies peroxiredoxins based on the number and location of conserved redox-sensitive cysteine residues in the peroxiredoxin [15]. Most peroxiredoxins have two conserved redox-sensitive cysteine residues—a peroxidatic cysteine that directly reduces various peroxide substrates and a resolving cysteine that can regenerate the peroxide reduction activity of the peroxidatic cysteine [16]. This class of peroxiredoxins is named typical or two-cysteine (2-Cys) peroxiredoxin. In typical peroxiredoxins, the peroxidatic and resolving cysteines are located on two different molecules [17]. Thus, typical peroxiredoxins usually work as homodimer formed via a stable inter-subunit disulfide bond (Figure 1). In contrast, both peroxidatic and resolving cysteines for the peroxidase reaction are on the same peroxiredoxin molecule for atypical peroxiredoxins. It explains why atypical peroxiredoxins usually function monomerically. The third class of peroxiredoxins is one-cysteine (1-Cys) peroxiredoxins that only contain the peroxidatic cysteine but no resolving cysteine [18]. The disulfide bridge formed after oxidation can be reduced by electron donors to restore the peroxidatic activity. While most of the peroxiredoxins use thioredoxin as their electron donor, certain isoenzymes of peroxiredoxin were reported to use glutaredoxins, cyclophilins, glutathione, and ascorbic acid as their electron donors [19,20,21,22].

## 4. Multiple Peroxiredoxins Are Commonly Found in an Organism

The importance of peroxiredoxins is reflected by their ubiquity and abundance. In budding yeast, there are five different peroxiredoxins [23]; in human and mouse, there are six different peroxiredoxins, respectively [24]. All of them show distinct but overlapping properties. Knockout or knockdown of individual peroxiredoxins in cells are often nonlethal and yield relatively mild or even no phenotype under standard growth conditions [25]. Increases in the amount of oxidized DNA and carbonylated protein, and reduction in the growth and survival rates of the cells after oxidative insults were usually observed [26]. The effects of peroxiredoxin deficiency became more obvious upon deletion of multiple peroxiredoxins and more prominent under-stress conditions [23,27]. For example, PRX1 KO mice have shorter lifespan, and developed age-dependent hemolytic anemias and various cancers [28]. PRX2 KO mice also have hemolytic anemia [29], but no cancer-related phenotype was reported. The amino acid sequence of mouse PRX1 and PRX2 proteins sharing 74% identity and 89% similarity and compensatory effects among the two peroxiredoxins were suggested. It was further assured by PRX1/PRX2 double knockout mice, which showed many novel abnormalities in addition to an aggravation of individual single knockout mice [30].

## 5. From an Antioxidant Enzyme to a Redox Signaling Regulator

It is generally agreed that the main function of peroxiredoxins is in defending against oxidative stress [23]. However, the antioxidant activity of peroxiredoxins is relatively weak [31]. For instance, it was observed that very low concentration of hydrogen peroxide (~100 μM) was sufficient to inactivate human PRX1 [32]. Indeed, the peroxidase activity of peroxiredoxins can be easily inactivated by hyperoxidation of their peroxidatic cysteine under mild oxidative stress. A special oxidoreductase sulfiredoxin is required to restore the peroxidase activity of a hyperoxidized peroxiredoxin [33]. ATP is required for sulfiredoxin to reduce sulfinic acid of peroxiredoxin back to thiol (Figure 1) [34].

Therefore, it was proposed that peroxiredoxins mainly function as a redox signaling regulator for regulating signaling pathways via the local concentration of free radical messengers and redox status of interacting proteins [35]. For example, peroxiredoxins can preserve the activity of PTEN by preventing oxidation of the intramolecular disulfide bond of PTEN [36]. Mechanistically, the intramolecular disulfide bond in PTEN is required for its function in inhibiting cell growth and proliferation by downregulating PI3K/AKT signaling pathway via dephosphorylating phosphatidylinositol (3, 4, 5)-trisphosphate (PIP3) to phosphatidylinositol (4, 5)-bisphosphate (PIP2). PRX1 can bind to and preserve the tumor-suppressive function of PTEN by preventing oxidation [36].

## 6. Role of Peroxiredoxin in Cell Cycle Regulation

The cell cycle machinery is dependent on the sequential expression of cyclins and activation of cyclin-dependent kinases (CDKs) that drives the cell cycle transitions from one to the next phase. Many cell-cycle regulatory proteins have redox-sensitive motifs [37]. It implies the possibility to regulate cell cycle by redox-dependent signaling pathways. It was demonstrated that intracellular concentration of H_2_O_2_ increases at G1–S phase transition, peak at mitosis, and then decrease during mitotic exit. At late G1–S phase transition and during mitosis, the peroxidase activity of PRX1 is inactivated by cell-cycle-dependent kinase 1 (Cdk1) through phosphorylation at Tyr194, leading to increased endogenous ROS, which inhibits APC/C–Cdh1 activity. In addition, phosphorylated PRX1 can associate with centrosome and this association is required for proper mitotic progression in mammalian cells [38]. Mechanistically, phosphorylated PRX1 indirectly inactivates centrosome-bound phosphatases by oxidation [38]. The knockdown of PRX1 leads to G2/M blockade in pancreatic ductal adenocarcinoma (PDAC) cells [39].

Interestingly, knockdown of other peroxiredoxins such as PRX2, PRX3, and PRX6 can also induce cell cycle arrest in mammalian cells [40,41,42]. PRX2 knockout in primary dermal mesenchymal stem cells (DMSC) exhibited significant accumulation of G_0_/G_1_ cell [40], trophoblast cells with PRX3 knockdown exhibited prolonged G_0_/G_1_ phase [41], and knockout of PRX6 induced cell cycle arrest at G2/M in HepG2 hepatocarcinoma cells [42]. Whether cell cycle arrests are peroxiredoxin- and/or cell-type-specific remains to be further explored.

## 7. Moonlighting Function as Chaperone

As mentioned above, homodimer of peroxiredoxins is formed via formation of inter-subunit disulfide bridge upon oxidation. Interestingly, peroxiredoxin homodimers may assemble to form a toroidal complex of 8, 10, or 12 subunits. The change in their quaternary structure regulates their functional switch from peroxidase to chaperone [43]. These toroid structures can stack with one another to form high molecular weight (HMW) nanotube-like structures or dodecahedron [44]. The oligomeric states of peroxiredoxin are dependent on many factors such as redox state, pH, and post-translational modifications.

The first in vivo evidence to support that peroxiredoxins may function as a chaperone under physiological condition came from the yeast peroxiredoxins TSA1 and TSA2 [45]. Both TSA1 and TSA2 can protect citrate synthase from aggregation induced by heat, insulin β chain from DTT-induced precipitation and tsa1/tsa2 double mutant yeast from heat shock [45]. Unexpectedly, the TSA1 mutants lacking peroxidase activity still consistently conferred tsa1/tsa2 double mutant yeast with heat shock resistance, although the level of resistance was still lower than that observed for the wild-type yeast. This suggests that the ability of TSA1 to protect the tsa1/tsa2 double mutant yeast from heat shock is not exclusively due to its peroxidase activity. Indeed, earlier study demonstrated that peroxidase-inactive TSA1 mutant lacking the functional resolving cysteine was still able to rescue the zinc-deficiency-induced protein aggregation phenotype in yeast [46].

Mechanistically, hyperoxidated TSA1 is required for the recruitment of cytosolic molecular chaperone HSP70 and disaggregase HSP104 to misfolded and aggregated proteins [47]. The aggregate resolution is triggered by the ATP-dependent peroxiredoxin sulfinic acid reductase Srx1 [47] (Figure 1). Chaperones such as heat shock proteins (HSPs) are thought to play an essential role in preventing aging [48] and progression of various cancers [49]. It remains to be explored whether the chaperone function of peroxiredoxins plays any role in senescence and tumorigenesis.

The roles of serine or threonine in the active site of typical 2-Cys peroxiredoxins on hyperoxidation susceptibility and on chaperone activity were reported recently [50]. 2-Cys peroxiredoxins containing serine at their active sites are more resistant to hyperoxidation and are more stable as decamers than those containing threonine [50]. Serine is more commonly seen in the active sites of bacterial 2-Cys peroxiredoxins than in eukaryotic peroxiredoxins [50]. That explains why bacterial peroxiredoxins display increased resistance to hyperoxidation, thermal resistance, and chaperone activity [50]. It may imply that the primary function of PRXs in lower prokaryotes is as peroxidase but, in higher eukaryotes, shifts to chaperone during evolution.

## 8. From Tumor Suppressor to Oncoprotein

Peroxiredoxins were firstly suggested to be tumor suppressors by inhibiting the oncoprotein c-Abl in 1997. PRX1 inhibits the tyrosine kinase activity of the proto-oncoprotein c-Abl by binding with the Src Homology 3 (SH3) domain of c-Abl leading to a cytostatic effect [51]. It was further reported that PRX1 also binds to the transactivation domain of the oncoprotein c-Myc and inhibits the expression of c-Myc target genes [52]. In addition, the tendency to have cancers was observed to rise in PRX1 knockout (KO) mice [28]. The high malignancy phenotype of PRX1 KO is due to increased sensitivity to oxidative DNA damage and abnormalities in their natural killer (NK) cells [28]. Later study demonstrated that PRX1 is one of the most prominently induced mRNAs in activated human NK cells [53] and supports the survival and antitumor activity of NK cells, especially under oxidative stress condition [54]. Therefore, on one hand, increased sensitivity to DNA oxidative damage in PRX1 KO mice increases the risk of many types of cancer. On the other hand, the NK cells in PRX1 KO mice fail to perform their antitumor immunity.

In addition, there is evidence indicating that peroxiredoxins suppress mutations by an oxidant-mediated DNA damage independent mechanism. Loss of TSA1 enhances mutation rates through elevation of deoxyribonucleotide triphosphate (dNTP) levels in yeast [55], and overexpression of TSA1 can rescue the mutator phenotype [56]. Constitutively high dNTPs concentration leads to mutagenesis by promoting polymerase slippage and impairing the polymerase proofreading activity [57,58]. TSA1 and TSA2 may limit dNTP synthesis by inhibiting overall ribonucleotide reductase activity [56,59]. It remains to be explored whether higher eukaryotic peroxiredoxins play any role in maintaining dNTPs pool.

Interestingly, human PRX2 can protect genome integrity by coupling fluctuations of dNTP biogenesis with DNA replication fork speed [60]. PRX2 can bind the fork accelerator TIMELESS and prevents the displacement of TIMELESS from the replisome [60]. Ribonucleotide reductase (RNR) is an essential enzyme that produces dNTPs for genome replication. When RNR catalyzes the reduction of ribonucleoside 5′-diphosphates (NDPs) into 2′-deoxyribonucleoside 5′-diphosphates (dNDPs) [61], ROS elevates in the microenvironment will disrupt the association of TIMELESS and chromatin via oxidizing PRX2 and leading to slowing down of replication fork progression [60].

However, in most circumstances, cancer cells are known to express high levels of peroxiredoxins [62]. Many mechanisms of peroxiredoxin in tumorigenesis are proposed. In brief, peroxiredoxins mainly act as a general cell survival enhancer not only for normal cells but also for cancer cells. Knockdown of peroxiredoxins suppresses their tumorigenic, metastatic, migrating, and invasive induction capacities. Overexpression of peroxiredoxins enhances tumorigenicity and associates with the development of chemoresistance [63] and poor prognosis in cancer patient [64]. Another mechanism is that PRX1 promotes carcinogenesis via induction of vascular endothelial growth factor (VEGF) expression [65]. That is why the approach of inhibiting peroxiredoxins was suggested for treatment of cancers [66].

## 9. Roles of Peroxiredoxins in Aging

It was reported that lack of active peroxiredoxins accelerates the aging process in various organisms—from yeast to mammals [25]. The evidence include the following: (1) decrease the overall expression level of peroxiredoxin in various cell types and tissues [67]; (2) increase the percentage of inactivated peroxiredoxin by overoxidation; (3) various aging-related phenotypes in knockout and overexpressing peroxiredoxins can be found in various mouse models. Table 1 summarizes the basic information of mammalian peroxiredoxins and age-associated phenotypes in respective knockout/transgenic mice.

More detailed mechanisms of peroxiredoxin in the oxidative stress theory of aging were proposed recently. For examples, p16 is a tumor suppressor that can push cells to enter senescence by preventing the phosphorylation of retinoblastoma (Rb) protein by the cyclin-dependent kinases CDK4 and CDK6 [74]. Loss of PRX1 can induce cellular senescence by inducing p16 expression in mouse embryonic fibroblast (MEF) [75]. It was also reported that PRX2 and PRX4 can inhibit the p16 signaling pathway by reducing the level of oxidative stress in chondrocytes [76] and ovarian aging in mice [77].

Interestingly, PRX1 can also prevent mitochondrial rupture via direct interaction with a crucial regulator of ROS level involved in aging dysfunction p66Shc [78]. p66Shc is an important ROS-sensitive probe that translates oxidative stress signals into mitochondrial apoptosis [79]. Genetic deletion of p66Shc in mice increased their lifespan and alleviated age-related pathologies [80,81].

Telomere shortening is a well-known hallmark of aging. Although the dynamics between oxidative stress and telomere shortening remain incompletely known [82], recent studies reported that PRX1 dampens telomere shortening [83,84,85]. PRX1 is enriched in telomeric chromatin, and this counteracts with ROS-induced telomere damage [84]. PRX1 KO cells accumulate telomeric single-strand DNA breaks after oxidative damage, which leads to rapid telomere shortening [84]. In addition, PRX1 cooperates with MTH1 to prevent the accumulation of oxidized guanine nucleotides such as 8-oxoguanine that inhibit telomerase when located at the 3′ ends of telomeric substrates [83,85].

In addition to PRX1, the role of PRX3 in aging was explored in a recent study [86]. Previous studies already demonstrated that PRX3 is essential for maintaining mitochondrial function by maintaining mitochondrial redox homeostasis [87,88]. This study demonstrated that the expression level of PRX3 is regulated by an age-associated glucose-responsive transcription factor MondoA in two in vitro senescence models—DNA-damage-induced senescence by doxorubicin-treated human retinal pigment epithelial cells and replicative senescence by TIG-3 cells [86]. The expression level of MondoA decreases with age that drives cellular senescence by impaired mitochondrial homeostasis through the expression of PRX3 [86]. In agreement with this finding, muscle-specific overexpression of PRX3 can attenuate contractile dysfunction and muscle atrophy in a SOD1 KO mouse model of accelerated sarcopenia by improving mitochondrial function [71].

## 10. Peroxiredoxins as Damage-Associated Molecular Patterns (DAMPs)

In response to stress and injury, certain endogenous molecules may be released or secreted from cells or exposed on the cell surface. These molecules are named damage-associated molecular patterns (DAMPs). DAMPs can trigger sterile inflammation via various innate immune receptors for tissue repair and regeneration. Efficient clearance of DAMPs is required to resolve inflammation. DAMPs can also lead to the development of many inflammation-related diseases, such as metabolic disorders, neurodegenerative diseases, autoimmune diseases, and cancer resulting from dysregulated sterile inflammation [89]. According to the “DAMP hypothesis” of aging and cancer published on Ageing Res Rev in 2015, increased stressors, especially oxidative stress, lead to DAMPs translocating and releasing into the extracellular space [90]. Loss of intracellular DAMPs increases genomic instability, epigenetic alteration, telomere attrition, reprogrammed metabolism, and impaired degradation system, whereas increased extracellular DAMPs cause excessive inflammation and immune injury [90]. Extracellular peroxiredoxins are now recognized as one of the proinflammatory factors, as they function as DAMPs for nervous system injury [91] and liver damages [92]. Mechanistically, in general, peroxiredoxins released from necrotic cells may bind to TLR2 and/or TLR4 to increase secretion of inflammatory cytokines by immune cells that aggravate ischemic stroke [91] and liver injury [92] (Figure 1).

Indeed, previous studies showed that peroxiredoxins from parasites may act as pathogen-associated molecular patterns (PAMPs) by triggering a proinflammatory response via binding to TLR4 receptor. The peroxiredoxins from parasites also protect the parasites from ROS/RNS produced by the host immune system [93]. Therefore, it seems that intracellular peroxiredoxins have protective functions against cellular stresses and extracellular peroxiredoxins are potentially harmful via inflammation. It is further confirmed by the fact that intraventricular injection of PRX2 alone caused hydrocephalus, ventricular wall damage, activation of macrophages, and an accumulation of neutrophils in rats [94]. 

## 11. Peroxiredoxin 6 with Particular Interest

Human PRX6 is a 224 amino acid protein with molecular weight 25 kDa. It has several characteristics setting it apart from other peroxiredoxin family members [95]. Firstly, as mentioned above, PRX6 is a 1-Cys peroxiredoxin that only have the peroxidatic cysteine but no resolving cysteine. Secondly, PRX6 uses glutathione instead of thioredoxin as an electron donor to restore its peroxidase activity. Thirdly, as demonstrated by the cyanobacterium Anabaena PRX6 (AnPRX6), it can form an asymmetric homodimer but not higher-order oligomers, and the dimer of AnPRX6 displays chaperone activity [96]. Fourthly, PRX6 has other moonlighting enzymatic functions, namely, phospholipid hydroperoxides [97], phospholipase A2 (PLA2) [98], and lysophosphatidylcholine acyltransferase (LPCAT) activities [99]. Due to the versatility of PRX6, it was suggested that PRX6 plays an important role in repairing oxidized cell membranes [100]. Activation of cell membrane repair machinery proves advantageous for survival of cells, particularly for cancer cells as they need robust repair capacity to cope with recurring injuries during navigation through dense extracellular matrix [101]. Therefore, it has been proposed that the peroxidase activity of PRX6 can promote the formation of metastatic colonies by stimulating cancer cell proliferation, whereas the PLA2 activity of PRX6 contributes to metastasis by enhancing the invasiveness of cancer cells [102]. Finally, PRX6 can suppress tumor necrosis factor-related apoptosis-inducing ligand (TRAIL)-induced death-inducing signaling complex (DISC) formation and, subsequently, caspase activation [103]. Death effector domain (DED) is a prototypical protein interaction domain that functions predominantly in the regulation and execution of programmed cell death and, additionally, in the control of cell proliferation. Among all PRXs, only PRX6 interacts with the DED of caspase-8 and caspase-10 as demonstrated by in vitro binding assays [103].

## 12. PRX6 and Senescence-Associated Secretory Phenotype

In most situations, senescent cells are locked into a cell-cycle arrest by the senescence program. It prevents the spread of damage to the next generation of cell and precludes potential malignant transformation [104]. Multiple types of stimulations, such as chemotherapy and radiation, can induce senescent cells to secrete high levels of interleukins, inflammatory cytokines, proteases, and growth factors, which are collectively referred to as senescence-associated secretory phenotype (SASP) [104]. A recent study using ionizing radiation-induced senescence (IRIS) cell model demonstrated that PRX6 is an important contributor to protection against oxidative-stress-induced cell death in senescent cells by attenuation of senescence-associated proinflammatory cytokine IL-6 secretion [105]. SASP can either promote or inhibit cancer, depending on the SASP composition. In addition, IL-6 is the most prominent cytokine of the SASP. PRX6 silencing decreased expression of extracellular matrix proteins including IL6 in IRIS hTERT-RPE-1 cells [105].

## 13. Closing Remarks and Future Perspectives

Peroxiredoxins are multifunctional enzymes that can function as redox signaling regulators, chaperones, and proinflammatory factors. Their peroxidase and chaperone activities are regulated by changing the redox states of their active center cysteine and switching between low- and high-molecular-weight species. Regulation of peroxiredoxin activities is not only crucial for the survival of normal cells, but also for senescence and carcinogenesis. Various inhibitors against peroxiredoxins have been developed for treatment cancers and peroxiredoxin-related diseases (summarized in Table 2).

As the peroxidase activity of PRXs is dependent on a cysteine situated in a highly conserved motif but not on any cofactor, it is a great challenge to develop their specific inhibitors. Indeed, some of the inhibitors listed in Table 2 are not specific for peroxiredoxins. For example, adenanthin also inhibits enzymes of the thioredoxin–thioredoxin reductase (TRXR-TRX) system [106]. Pentagamavunon-1 (PGV-1) can inhibit cancer cell growth via suppressing angiogenic factors cyclooxygenase-2 (COX-2) and vascular endothelial growth factor (VEGF) expressions and NF-κB activation [107]. Frenolicin B also targets glutaredoxin 3 to trigger ROS/4E-BP1-mediated antitumor effects [108].

**Table 2:** List of peroxiredoxin inhibitors.

Target	Inhibitor	Action	Result	Model	Reference
PRX1	Pentagamavunon-1 (PGV-1), a curcumin analog	Bound to several ROS-metabolizing enzymes, including PRX1	Induced G2/M cell cycle arrest and cell senescence	Highly metastatic breast cancer cell line, the 4T1 cells	[109]
PRX1	Epo-C12, a synthetic derivative of epolactaene	Inhibit PRX1 peroxidase but not its chaperone activity.	Exerted an apoptotic effect	BALL-1 cells	[110]
PRX1	Ferulic Acid Amides	Inhibit peroxidase activity	Improved hyperglycemia and hyperlipidemia	Streptozotocin-nicotinamide-induced diabetic rats	[111]
PRX1	Frenolicin B	Target the active cysteine residues	Increased levels of intracellular ROS to induce apoptosis and suppress tumor growth	Nude mice bearing established HCT116 or DLD-1 colorectal cancer xenografts	[108]
PRX2	Conoidin A	Inhibit peroxidase activity	Inhibited the growth of the 5-FU-resistant gastric cancer SNU620 cells	5-FU-resistant SNU620 cells	[112,113]
PRX1 /PRX2	Adenanthin, a diterpenoid isolated from the leaves of Rabdosia adenantha	Inhibit enzymes of the PRX-related chain including thioredoxin and thioredoxin reductase	Induced differentiation of acute promyelocytic leukemia (APL) expresses tumor growth in vivo and prolongs survival	Mouse APL models	[114]
			Killed these malignant liver cells in vitro and xenografts	SMMC-7721 cells were transplanted into BALB/c nude mice	[115]
			Impaired the spontaneous and antibody-dependent NK cell cytotoxicity against cancer cells	K562 and Raji cell lines, primary human NK cells	[53]
PRX1 /PRX2	Parvifoline AA	Inhibit peroxidase activity	Activated the ROS/ERK axis and the immunogenicity of hepatocellular carcinoma toward natural killer cells.	Hepa1-6 mouse allograft model	[116]
PRX6	9 amino acid peptide named as PIP-2	Inhibit of PRX6 Phospholipase A 2 Activity	Protected against Lung Injury	Mouse Model of Ventilator-Induced Lung Injury	[117]

In addition, the importance of inhibiting peroxiredoxins in cancer treatments remains to be explored as most of the relevant studies remain at the preclinical level. Clinical studies are required to establish the safety and effectiveness of treating cancers by inhibiting peroxiredoxins. As multiple peroxiredoxins are expressed at high level in most cells in our body, the potential side effects shall be considered.

## Figures and Tables

**Table 1:** List of mammalian peroxiredoxins and their age-associated phenotypes in mouse.

Isoenzyme.	Type	Main Subcellular Localization	Age-Associated Phenotypes in Mouse Model	Reference
PRX1	Typical 2-Cys	Cytosol	Shorter lifespan Develop age-dependent Hemolytic anemias and various cancers in PRX1 KO mice	[28]
PRX2	Typical 2-Cys	Cytosol	Aggravates aging-induced insulin resistance and declines muscle strength in PRX2 KO mice	[68]
			Aggravates age-related ovarian failure in PRX2 KO mice	[69]
PRX3	Typical 2-Cys	Mitochondria	Reduces the severity of age-related osteoarthritis in PRX3 overexpressing mice	[70]
			Reduces age-related muscle atrophy and weakness in PRX3-overexpressing mice	[71]
PRX4	Typical 2-Cys	Endoplasmic reticulum, extracellular space	PRX4 deficiency was associated with mortality in adult and aged mice	[72]
PRX5	Atypical 2-Cys	Cytosol, mitochondria, and peroxisomes	No age-associated phenotype reported yet	N.A.
PRX6	1-Cys	Cytosol, lysosomes	Decreases fertility in PRX6 KO mice	[73]
